# Tolerance of High Oral Doses of Nonradioactive and Radioactive Caesium Chloride in the Pale Grass Blue Butterfly *Zizeeria maha*

**DOI:** 10.3390/insects10090290

**Published:** 2019-09-09

**Authors:** Raj D. Gurung, Wataru Taira, Ko Sakauchi, Masaki Iwata, Atsuki Hiyama, Joji M. Otaki

**Affiliations:** 1 The BCPH Unit of Molecular Physiology, Department of Chemistry, Biology and Marine Science, University of the Ryukyus, Okinawa 903-0213, Japan (R.D.G.) (W.T.) (K.S.) (M.I.) (A.H.); 2 Instrumental Research Center, University of the Ryukyus, Okinawa 903-0213, Japan; 3 Department of International Agricultural Development, Faculty of International Agriculture and Food Studies, Tokyo University of Agriculture, Tokyo 156-8502, Japan; 4 Japan Butterfly Conservation Society, Tokyo 140-0014, Japan

**Keywords:** caesium chloride, field effect, Fukushima nuclear accident, internal exposure, pale grass blue butterfly, radioactive caesium, toxicology, *Zizeeria maha*

## Abstract

The biological effects of the Fukushima nuclear accident have been examined in the pale grass blue butterfly, *Zizeeria maha* (Lepidoptera: Lycaenidae). In previous internal exposure experiments, larvae were given field-collected contaminated host plant leaves that contained up to 43.5 kBq/kg (leaf) of radioactive caesium. Larvae ingested up to 480 kBq/kg (larva), resulting in high mortality and abnormality rates. However, these results need to be compared with the toxicological data of caesium. Here, we examined the toxicity of both nonradioactive and radioactive caesium chloride on the pale grass blue butterfly. Larvae were fed a caesium-containing artificial diet, ingesting up to 149 MBq/kg (larva) of radioactive caesium (^137^Cs) or a much higher amount of nonradioactive caesium. We examined the pupation rate, eclosion rate, survival rate up to the adult stage, and the forewing size. In contrast to previous internal exposure experiments using field-collected contaminated leaves, we could not detect any effect. We conclude that the butterfly is tolerant to ionising radiation from ^137^Cs in the range tested but is vulnerable to radioactive contamination in the field. These results suggest that the biological effects in the field may be mediated through ecological systems and cannot be estimated solely based on radiation doses.

## 1. Introduction

Field observations of the possible biological effects of radioactive contamination after the Fukushima nuclear accident have been reported in various organisms, including bird and arthropod populations [1,2], gall-forming aphids [3], Japanese monkeys [4,5,6], barn swallows [7], goshawks [8], rice plants [9,10], fir trees [11], red pine trees [12], and intertidal species populations, including the rock shells [13]. Similarly, the possible changes induced by the nuclear accident have been reported in cattle at the biochemical level [14] and in pigs at the gene expression level [15]. Additional cases include DNA damage in bovine lymphocytes [16], enhanced spermatogenesis [17], and chromosomal aberrations [18,19] in large Japanese field mice. Recently, an increase in the human perinatal mortality in contaminated areas after the accident has been reported [20]. Changes in the genome-wide DNA methylation levels have been detected in plants from Chernobyl but not from Fukushima [21]. However, mammalian testes collected from bulls, bore, inobuta, and large Japanese field mice in the contaminated area did not reportedly show any noticeable abnormalities [22,23,24]. Furthermore, an external exposure experiment of mice using ^60^Co as the radiation source failed to detect an effect on the litter size and sex ratio at the exposure levels of the Fukushima nuclear accident [25]. Blood cell counts and thyroid glands have been normal in cattle in the contaminated area [26,27]. Striking morphological abnormalities of the aphids that were detected in the field [3] have been tested experimentally in the laboratory to result in minor physiological changes [28].

The pale grass blue butterfly, *Zizeeria maha* (Lepidoptera: Lycaenidae), is an organism that has well-coordinated field observations and laboratory replication experiments [29,30,31,32]. This butterfly has been monitored in the contaminated areas around Fukushima since May 2011, two months after the Fukushima nuclear accident [29]. Importantly, internal exposure experiments have been performed by feeding the field-collected contaminated host plant leaves, *Oxalis corniculata*, to larvae of the pale grass blue butterfly from Okinawa, the least contaminated locality in Japan [29,33,34,35]. The contaminated leaves were collected from localities of various contamination levels. As a result, the total abnormality rate (including the dead and abnormal individuals) was shown to fit a Weibull function curve or a power function curve in response to radioactivity concentrations of ^134^Cs and ^137^Cs (the most biologically significant radionuclides released from the Fukushima Dai-ichi Nuclear Power Plant) in the collected leaves [35]. The Weibull function is a sigmoidal curve that is known to describe a failure process of a mechanical or biological machine. The half abnormality dose from the Weibull function fit was 1.36 × 10^4^ Bq/kg (larva) [35]. These ecotoxicological results indicate that the pale grass blue butterfly is sensitive to ionising radiation or its associated contamination effects in the field.

The United Nations Scientific Committee on the Effects of Atomic Radiation (UNSCEAR) positively commented on our studies, including the internal exposure experiments [36]. However, UNSCEAR reserved acceptance of our conclusions because insects are known to be relatively resistant to radiation exposure [36]. The pale grass blue butterfly may be an exceptional case, but there is a possibility that the field-based results may be different from the laboratory-based results under controlled experimental conditions [37,38,39,40,41,42].

Our internal exposure experiments were indeed designed to reflect the field contamination as a whole, and our results do not necessarily indicate radiation sensitivity under laboratory conditions. Although the toxicological data on the contaminated leaves are very important for understanding the real-world phenomena in Fukushima, it may not indicate the toxicology of radiocaesium itself under controlled laboratory conditions.

To resolve this issue, the pale grass blue butterfly was subjected to toxicological tests using commercially available radioactive caesium chloride in the present study. Although the relevance of this experiment with the real-world biological effects of the Fukushima nuclear accident is obscure, this experiment makes it possible to compare the field-based ecotoxicological data of contaminated leaves with the laboratory-based toxicological data of radioactive caesium in the same species to highlight their differences, if there are any. Additionally, the toxicity of nonradioactive caesium chloride was tested to examine the chemical effects of caesium.

Here, we first developed an improved version of an artificial diet that was less dependent on *Oxalis* leaves compared to the previous diet [43]. Using this newly developed artificial diet, termed AD-FSI-112, we successfully reared larvae with nonradioactive (cold) and radioactive (hot) caesium chloride and tested for their toxicological effects on the pale grass blue butterfly under controlled laboratory conditions.

## 2. Materials and Methods

### 2.1. Butterfly and Its Host Plant

Throughout this study, the pale grass blue butterfly *Zizeeria maha* (Kollar, 1844) was used. We collected both male and female adult individuals from the campus of the University of the Ryukyus and its surroundings on Okinawa-Jima Island. We confirmed that these adult butterflies did not have any noticeable morphological abnormalities. Eggs were collected from these females according to the standard procedure [43]. The larval natural host plant *Oxalis corniculata* was collected from the field on Okinawa-Jima Island. No permissions were required to collect the butterflies and plants from the wild, to rear this butterfly in the laboratory, or to perform experiments with this butterfly in Okinawa, Japan.

### 2.2. Preparation of the Artificial Diet

To test the radioactive and chemical (nonradioactive) toxicity of ^137^Cs in the pale grass blue butterfly, an artificial diet was required. Previously, we developed an artificial diet for the pale grass blue butterfly [43], but the previous diet was time-consuming to make and required a relatively large amount of *Oxalis* leaves. We sought an artificial diet that demanded fewer *Oxalis* leaves to lessen its possible unknown contribution to biological effects and to make the preparation easier without harming any performance as a viable diet.

There may still be room for further improvement in the new diet. As discussed previously [43], larvae smaller than 3–5 mm (the late third instar stage) could not eat the diet well. It has been reported that oxalic acid present in *Oxalis* leaves is likely the main stimulant for larvae to initiate eating behaviour in the pale grass blue butterfly [44]. Addition of oxalic acid to an artificial diet could provide further opportunities to explore more efficient diets. However, we did not use it in the present study because our preliminary tests were not very positive.

**Figure A1:**
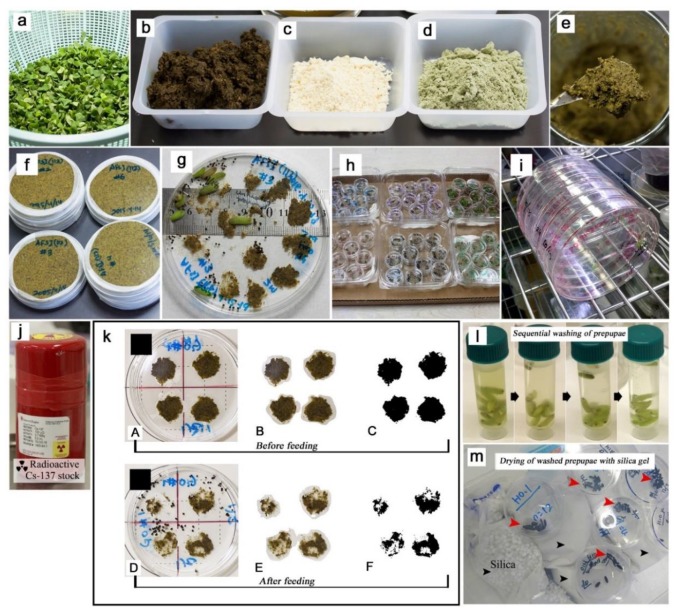
Materials and methods used in this study. (**a**) Separated and washed *Oxalis* leaves. (**b**) Fresh *Oxalis* leaf paste. The dry weight of this paste is considered one part. (**c**) Soy powder (one part) (**d**) Insecta F-II (two parts). (**e**) Mixing the fresh *Oxalis* paste, soy powder, and Insecta F-II in a glass cup using a spoon. (**f**) Plated artificial diet in Petri dishes. Dishes were labelled and sealed. (**g**) Feeding larvae an artificial diet in a 90-mm Petri dish. This panel shows a preliminary experiment to test the quality of the diet. Only 35-mm Petri dishes were used for feeding in the toxicological experiments. (**h**) The larval rearing system. In a plastic container, 35-mm Petri dishes were confined. (**i**) Isolated pupae in 90-mm Petri dishes. (**j**) Original supply of ^137^CsCl (3.7 MBq in 0.5 mL). (**k**) Image analysis for areas of the artificial diet consumed by the larvae. [A–C] Images before feeding; [D–F] Images after feeding. From the original pictures [A,D], the diet areas and their surroundings were cut out [B,E], and the diet areas were further isolated by applying a darkness threshold of 133 using Adobe Photoshop. Black squares are reference scale areas of 1.0 cm^2^. Areas were then calculated using ImageJ. (**l**) Sequential washing of the prepupae (light green objects) with deionised water to remove the artificial diet from their bodies before measuring the radioactivity concentrations of ^137^Cs. (**m**) Drying of the washed prepupae with silica gel (black arrowheads) for measuring the radioactivity concentrations of ^137^Cs. Dried prepupae were black and hard (red arrowheads).

Fresh leaves of *Oxalis corniculata* were collected from the field in Okinawa–Jima Island and, within one day after collection, plucked (separated from the stem) and washed thoroughly with tap water followed by deionised water (Figure A1a). Extra water on the leaves was eliminated. The leaves were then ground into fine paste manually (Figure A1b) and mixed with other ingredients. AD-FSI-112 (artificial diet containing fresh leaves, soy powder, and Insecta at a ratio of 1:1:2) contained one part of the dried equivalent of fresh leaves (Figure A1b), one part of the soy powder (Figure A1c), and two parts of Insecta F-II (Nosan Corporation, Yokohama, Japan) (Figure A1d). The dry weight of these four parts × 2.6 was the amount of water added in millilitres. The amount of the vitamin complex solution was the *Oxalis* dry weight × 5 in microlitres. The amount of linseed oil added was the amount of the four parts × 0.005 in microlitres. According to Hiyama et al. (2010) [43], the following eight ingredients were mixed, and deionised water was added to a final volume of 500 μL to make the vitamin complex solution: corn starch 122.9 mg (Sigma-Aldrich, Saint Louis, MO, USA), D-biotin 0.05 mg (Kanto Chemical, Tokyo, Japan), D-pantothenic acid hemicalcium salt 0.5 mg (Sigma-Aldrich), folic acid 0.05 mg (Sigma-Aldrich), nicotinamide 0.5 mg (Kanto Chemical), pyridoxine hydrochloride 0.25 mg (Sigma-Aldrich), riboflavin 5’-monophosphate salt dehydrate 0.25 mg (Sigma-Aldrich), and L-carnitine inner salt 0.25 mg (Sigma–Aldrich). Thiamine hydrochloride, used in Hiyama et al. (2010) [43], was not added in this study.

According to manufacturer’s specification, Insecta F-II is a mash-type powder-based food for various lepidopteran and coleopteran insect larvae containing chlorella powder, defatted soybean, starch, sugar, cellulose, agar, citric acid, vitamin complex, minerals, preservatives, and antibiotics. The nutrient content of Insecta F-II is reported from the manufacturer as follows: water (10.0% or less), protein (23.0% or more), fat (3.0% or more), fibre (16.0% or less), and salt (16.0% or less).

All ingredients except the vitamin complex solution were mixed in a large glass cup with long-handled spoons (Figure A1e). The mixture was then heated using a microwave (500 Watt, 15–20 s). To make it homogeneous, the mixture was once again mixed thoroughly at temperatures below 60 °C, and then the vitamin complex solution was added. To avoid water loss and possible contamination, the glass cup was covered with plastic wrap throughout the preparation process. Using a small sterile spatula, the artificial diet was layered (approximately 2 mm thickness) in 90-mm Petri dishes. Once the diet was evenly layered in the Petri dishes, they were sealed airtight with parafilm (Figure A1f) and placed upside down inside an airtight container. The whole containers were stored in a refrigerator (4–5 °C) for at least 24 h before use. A fresh artificial diet was prepared for each batch of rearing experiments.

### 2.3. Egg Harvesting and Larval Rearing

The overall procedures for egg harvesting and larval rearing were similar to the previous study [43]. Three cages for egg collection were set simultaneously with a total of 6–9 females to collect a sufficient number of eggs in a few days to reasonably synchronise larval growth. Eggs from several females were mixed together to lessen the genetic bias. Throughout the rearing process, 16 h light: 8 h dark conditions were maintained. Until the late third instar stage, the larvae were reared with a natural diet (*Oxalis* leaves) in a plastic container at an ambient temperature of approximately 27 °C. Later, the artificial diet AD-FSI-112 was given to larvae.

To test the quality of AD-FSI-112, a 90-mm Petri dish with several patches of the artificial diet was used (Figure A1g). For the caesium toxicity tests, four patches of the artificial diet were placed in a 35-mm Petri dish (Figure A1k). In this case, each patch contained 50 ± 1 mg of the artificial diet; in total, one Petri dish contained approximately 200 mg. To avoid water evaporation during the diet plating, the diet was kept in an airtight plastic container with a cluster of wet tissue paper. A 5 μL drop of the test solution (i.e., cold or hot caesium chloride solution of various concentrations) was placed on each patch of the artificial diet. Thus, 20 μL in total was used per Petri dish.

Late third instar larvae were used for the artificial diet feeding experiments. Larvae of similar sizes were selected for rearing. Only three larvae were confined per Petri dish. Then, five or six Petri dishes (not airtight) with the same dosage of caesium were confined in an airtight plastic container together with wet tissue paper (Figure A1h). The plastic container was open at least once a day for aeration, and the wet tissue paper was replaced every other day. In the case of feeding hot caesium, the rearing containers of each dosage group were isolated by standing lead blocks around them. This experimental setup avoided external irradiation from adjacent groups but cannot avoid external irradiation from the artificial diet that the larvae were eating and from other Petri dishes in the same container. An individual larva is small compared to a human body; therefore, the distinction between external and internal irradiation may not be relevant when larvae were on the hot diet. Dosimetric calculations for the larvae may be expected, but it is beyond the scope of this study.

The light intensity during the feeding of the artificial diet was set relatively low to avoid a possible temperature increase and the evaporation of water inside a Petri dish. Larvae were transferred to a new Petri dish containing new patches of the artificial diet every other day, but large fourth instar larvae were transferred to a new Petri dish every day. After pupation, each pupa was transferred to a 90-mm Petri dish with high light intensity (Figure A1i).

### 2.4. Nonradioactive and Radioactive Caesium Chloride Preparation

Nonradioactive caesium chloride (Sigma-Aldrich) was dissolved in deionised water to produce various concentrations of CsCl solution. First, the highest concentration solution was made by mixing 1.266 g CsCl with deionised water to produce a 32-mL solution. This solution contained 1.000 g of Cs, and the Cs concentration was 235.1 mM. Then, 0.1 M NaOH and 0.1 M HCl were added to make the ionic species and concentrations of the nonradioactive solution similar to those of the radioactive solution. This solution was called C11. The C11 solution was serially diluted to make a 1/10 concentration of the previous solutions. C11, C9, C7, C6, C5, and C4 were used for the feeding experiments. All solutions were maintained between pH 6 and pH 7. The control solution was termed C0, which was deionised water.

All experiments with ^137^Cs were performed in the RI (radioisotope) facility of the University of the Ryukyus. Radioactive caesium chloride stock solution (^137^CsCl) at a concentration of 3.7 MBq in 0.5 mL of 0.1 M HCl (Figure A1j) was obtained from Eckert and Ziegler Isotope Products (Valencia, CA, USA) through the Japan Atomic Energy Agency (Tokai-Mura, Japan). Initially, the highest concentration solution was made by mixing a required amount of stock solution with 0.1 M NaOH and water. This solution was termed H6. The H6 solution was serially diluted to make a 1/10 concentration of the previous solution, producing H5, H4, H3, H2, and H1. All solutions were maintained between pH 6 and pH 7. The control solution was termed H0, which was deionised water.

### 2.5. Toxicological Outputs

The rearing results were evaluated by the pupation rate, the eclosion rate, the survival rate, and the forewing size. The pupation rate was defined as the number of larvae that successfully pupated divided by the total starting number of larvae used. The eclosion rate was defined as the number of pupae that successfully eclosed divided by the total number of pupae. The survival rate was defined as the number of individuals who successfully became adults divided by the total starting number of larvae used. All three rates were expressed as percentages. An adult individual that escaped from its pupal case was counted as surviving, irrespective of the morphological abnormality (i.e., wrinkled wings). Hiyama et al. [43] used a failure rate for evaluation of artificial diets, which included all dead pupae and abnormal adults. Adult individuals with wrinkled wings were rare, although not zero; therefore, the survival rate of Hiyama et al. [43] was converted from the failure rate in the present study. The right forewing size of successfully eclosed adults was measured from the base to the wing apex using a digital microscope SKII (Saitoh Kogaku, Yokohama, Japan). Adults with panda-type colour patterns [43] and wrinkled wings were included as individuals with successful eclosion in the present study, but they were not subjected to the forewing size measurements. Forewings with physical damage after eclosion were also excluded from measurements.

### 2.6. Image Analysis of the Artificial Diet Consumed

Using a camera stand that was built by ourselves to hold a Canon EFS-60 digital camera with an F2.8 macro lens (Canon, Tokyo, Japan) at a fixed height, photographs of the Petri dishes with the artificial diet were taken before and after feeding (Figure A1k(A,D)). The diet images were isolated (Figure A1k(B,E)), and the diet areas were made black by applying a darkness threshold of 133 after the convergence to greyscale mode using Adobe Photoshop (Adobe Systems, San Jose, CA, USA) (Figure A1k(C,F)). Areas of the diet patches before and after feeding were then calculated using ImageJ (National Institutes of Health, Bethesda, MD, USA), and the differences in the area were obtained. The area difference was then converted to the volume of the artificial diet consumed by a single larva. A single larva consumed 108.6 ± 73.4 mg (mean ± SD; *n* = 3) of the artificial diet on average from the late third instar stage until the prepupal stage.

### 2.7. Radioactivity Measurements of Prepupae

We used prepupae instead of larvae and pupae to measure the radioactivity retained in the body. This is because the larvae stopped eating and excreted the radioactive substance in the digestive tract before the prepupal stage but retained all parts of the larval body, including the larval cuticle. Moreover, prepupae are easy to handle because they do not move. For the radioactivity measurements, 10 to 19 prepupae per concentration level were collected and washed thoroughly with deionised water at least five times to completely remove hot diets on the surface of the prepupae (Figure A1l). After the washing stage, water droplets on the prepupae were completely removed, and the prepupae were frozen at −20 °C overnight. Dead prepupae were then transferred to an airtight container together with silica gels or similar desiccants. They were left for three weeks at ambient temperature until they were fully dried and became dark brown and hard (Figure A1m). Then, they were carefully crushed into a powder in a 2 mL tube. The weights of the empty tubes and those with the crushed prepupae were recorded to calculate the weight of the prepupae. To make a 1-mm sample height inside a well, 10–19 prepupae were used. The lids of the tubes were tightly sealed with parafilm. The radioactivity values of the sample were then measured using a germanium semiconductor device (Mirion Technologies (Canberra), Meriden, CT, USA) and analysed using Genie 2000 Gamma Analysis Software (Mirion Technologies (Canberra)). Measurements were carried out until the error rate reached less than 3%. The detection efficiency of ^137^Cs (662 keV) of 19.3% and the branching ratio of 85.1% were used for the calculations of the radioactivity concentrations of ^137^Cs on the day of measurements. Radioactivity measurements were carried out within a few months after the feeding experiment, and we confirmed that a dose decrease due to radioactive decay within a few months was insignificant (0.45% at most). Thus, radioactivity values on the day of measurements were used for subsequent analyses.

### 2.8. Statistical Analysis

The statistical software R (The R foundation for Statistical Computing, Vienna, Austria) and JSTAT (Sato, Yokohama, Japan) were used to perform the Kruskal–Wallis rank sum test, the Spearman correlation analysis, and the unpaired Student’s *t*-test.

## 3. Results

### 3.1. Quality Check of the Artificial Diet

**Figure 1:**
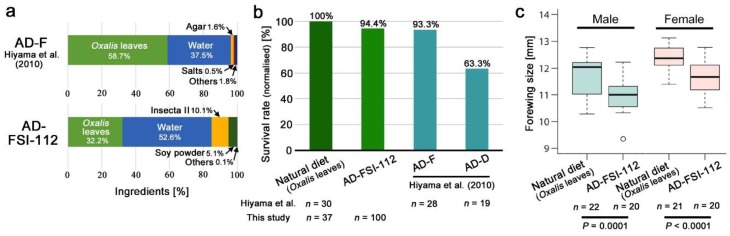
Evaluation of the artificial diet AD-FSI-112. (**a**) Ingredients of the artificial diets AD-F, according to Hiyama et al. (2010) [43], and AD-FSI-112. Ingredients are shown in weight proportions. (**b**) Survival rates (normalised). Data for AD-F and AD-D were obtained from Hiyama et al. [43]. (**c**) Forewing size. *p*-values are shown (Student’s *t*-test).

We first developed a new artificial diet, AD-FSI-112 (see Materials and Methods), and compared the ingredients with those of the previous diet, AD-F, in weight proportions [43]. Whereas *Oxalis* leaves composed 58.7% of the entire weight in AD-F, the leaves composed only 32.2% in AD-FSI-112, indicating a reasonable decrease in the leaf demand (Figure 1a). When the survival rate of larvae to adults using the natural diet (*Oxalis* leaves) was set at 100% for normalisation, the survival rate with AD-FSI-112 was 94.4% (for reference, the absolute number of the survived individuals was 71 out of 100), which was comparable to the diet previously described [43] (Figure 1b). On the other hand, the forewing size of the adults reared with the artificial diet AD-FSI-112 was significantly smaller than those with the natural diet (Student’s *t*-test; *t* = 4.2, *df* = 40, *p* = 0.0001 for males; *t* = 4.4, *df* = 39, *p* < 0.0001 for females) (Figure 1C). However, because this size decrease has been known from a previous study with no unfavourable effects in a rearing experiment, we decided to use AD-FSI-112 for the oral administration of caesium in subsequent experiments.

### 3.2. The Caesium Levels Tested

**Figure 2:**
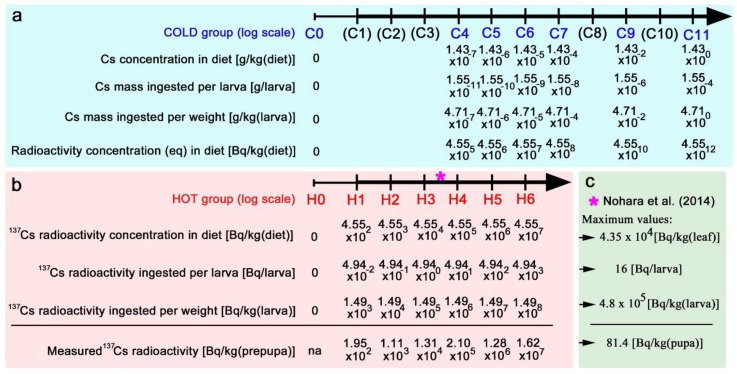
Caesium levels used in the present study. In a and b, numerical values were obtained from calculations based on the starting concentrations except for the last line in b. The amount of the artificial diet consumed was assumed to be 108.6 mg (see Materials and Methods). Larval weight was assumed to be similar to the weight and to be 0.033 g, from Nohara et al. [33]. (**a**) Nonradioactive (cold) caesium group. The radioactivity concentration equivalence was calculated based on the fact that 100 Bq of ^137^Cs is 31.2 pg, according to Shozugawa [45]. C1, C2, C3, C8, and C10 were not used in the present study. (**b**) Radioactive (hot) caesium group. H4, H5, and H6 correspond to C4, C5, and C6, respectively. An asterisk indicates the levels of the previous internal exposure experiments. The bottom lines are not calculated but are measured values. na: Not applicable. (**c**) Radioactivity concentrations from the previous internal exposure experiments. Data were obtained from Nohara et al. [33]. The bottom concentration is not calculated but is the measured value.

In this study, we used both nonradioactive (cold) and radioactive (hot) caesium chloride, and the caesium levels tested are summarised in Figure 2. For nonradioactive caesium (Figure 2a), the maximum level tested was defined as C11, the concentration of which was 1.43 g/kg (diet). The level decreased in a logarithmic scale down to C4, the concentration of which was 1.43 × 10^−7^ g/kg (diet). Based on the amount of the diet ingested by a larva, which was 108.6 mg (see Materials and Methods), we calculated the total mass of caesium ingested per larva, which was then converted to the amount of caesium ingested per kilogram (larva). We further calculated the concentrations of the nonradioactive caesium that were equivalent to the radioactivity concentrations of ^137^Cs in the diet [45].

For radioactive caesium (Figure 2b), the maximum level tested was defined as H6, the radioactivity concentration of which was 4.55 × 10^7^ Bq/kg (diet). The levels decreased on a logarithmic scale down to H1, the radioactivity concentration of which was 4.55 × 10^2^ Bq/kg (diet). Similar to the cold case, the radioactivity ingested per larva was calculated and converted to the radioactivity ingested per kilogram (larva). It is important to stress that the cold groups C4, C5, and C6 exactly corresponded to the hot groups H4, H5, and H6, respectively; they (e.g., C4 and H4) contained the same chemical amount of caesium. This allowed us to compare their differences to directly test the effects of radioactivity of ^137^Cs. To confirm that larvae indeed consumed ^137^Cs, radioactivity concentrations of prepupae were measured (Figure 2b).

The caesium levels above should also be compared with those of a previous study in which field-collected contaminated *Oxalis* leaves were used for internal exposure experiments [29,33] (Figure 2c). The maximum ^137^Cs radioactivity concentration in contaminated *Oxalis* leaves (wet) was 4.35 × 10^4^ Bq/kg (leaf), and larvae ingested 16 Bq in a previous study [33]. This was converted to 4.8 × 10^5^ Bq/kg (larva). These levels corresponded to the levels between H3 and H4 in the present study.

### 3.3. Nonradioactive Caesium Chloride

**Figure 3:**
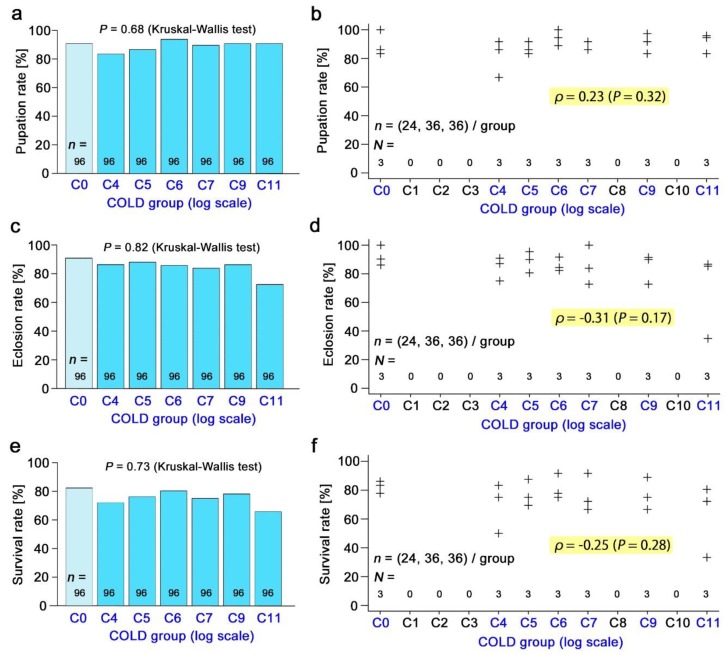
Effects of nonradioactive caesium chloride. In a, c, and e, all individual samples in the 3 trials were summed together, and the differences among the groups were examined using a Kruskal–Wallis test. In b, d, and f, the three trials were independently plotted, and a Spearman correlation analysis was performed. *N* indicates the number of groups. (**a**,**b**) The pupation rate (%). (**c**,**d**) The eclosion rate (%). (**e**,**f**) The survival rate (%).

**Figure 4:**
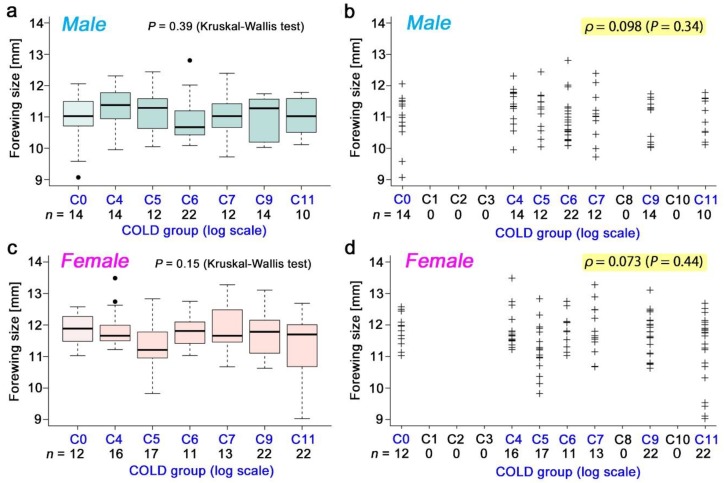
Forewing size of the individuals who consumed nonradioactive caesium chloride. In a and c, box plots were made, and the differences among groups were examined using a Kruskal–Wallis test. In b and d, individual sample data were plotted, and a Spearman correlation analysis was performed. (**a**,**b**) Male forewing size. (**c**,**d**) Female forewing size.

The pupation rate (Figure 3a,b), eclosion rate (Figure 3c,d), and survival rate (Figure 3e,f) in response to the nonradioactive caesium concentration were examined in a wide range up to C11. There was no significant difference in the Kruskal–Wallis test and no correlation in the Spearman correlation analysis. The male forewing size (Figure 4a,b) and the female forewing size (Figure 4c,d) were also examined, but no significant change was observed.

### 3.4. Radioactive Caesium Chloride

**Figure 5:**
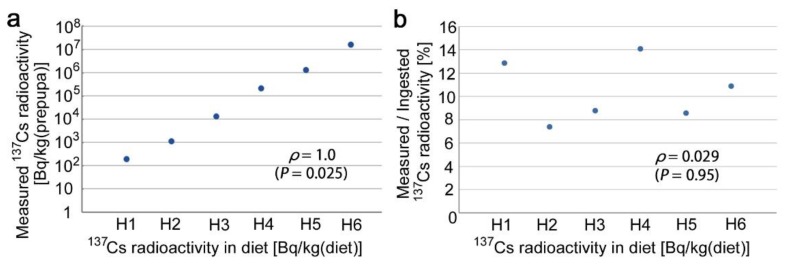
Caesium radioactivity in the prepupae. Spearman correlation coefficients and their associated *P*-values are reported. (**a**) Measured caesium radioactivity in the prepupae in proportion to that in the artificial diet. Both the *x*- and *y*-axes are on a logarithmic scale. (**b**) Percentage of the measured caesium radioactivity among the ingested caesium radioactivity plotted against the caesium radioactivity in the artificial diet. In this scatter plot, the *x*-axis is on a logarithmic scale.

The measured ^137^Cs radioactivity of the prepupae was proportional to the radioactivity in the diet (Figure 5a). The percentage of the measured radioactivity among the ingested radioactivity did not change much in response to the amount of radioactivity in the diet (Figure 5b). These data confirmed that the oral administration of ^137^Cs in larvae was successful over the range tested.

**Figure 6:**
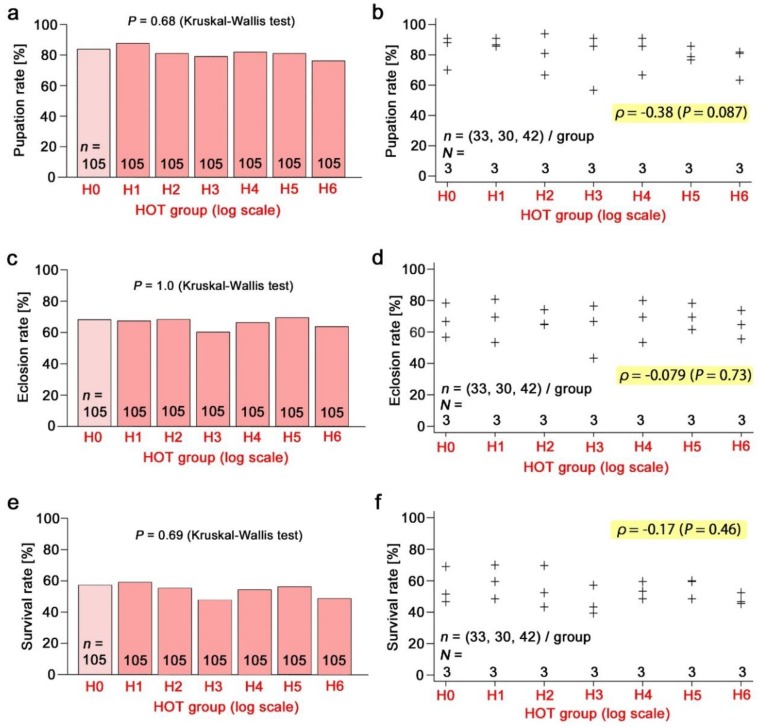
Effects of radioactive caesium chloride. In a, c, and e, all individual samples in the three trials were summed together, and the differences among the groups were examined using a Kruskal–Wallis test. In b, d, and f, the three trials were independently plotted, and a Spearman correlation analysis was performed. *N* indicates the number of groups. (**a**,**b**) The pupation rate (%). (**c**,**d**) The eclosion rate (%). (**e**,**f**) The survival rate (%).

**Figure 7:**
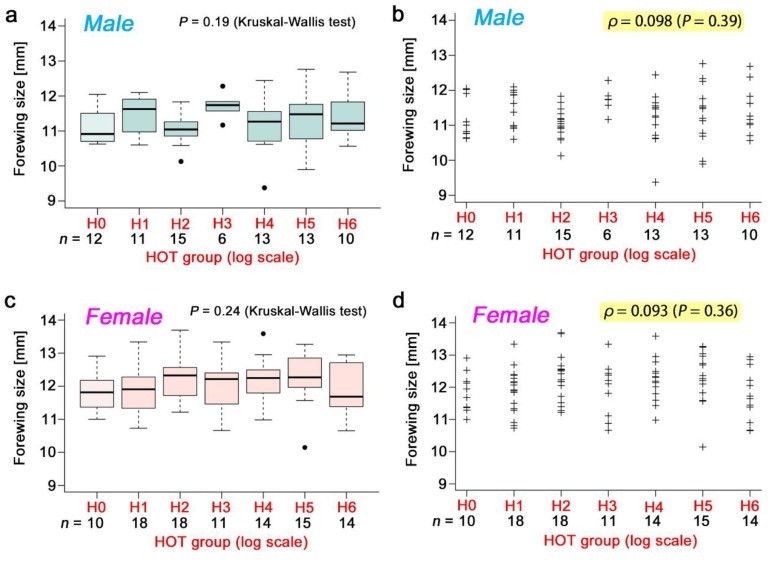
Forewing size of the individuals who consumed radioactive caesium chloride. In a and c, box plots were made, and the differences among groups were examined using a Kruskal–Wallis test. In b and d, individual sample data were plotted, and a Spearman correlation analysis was performed. (**a**,**b**) Male forewing size. (**c**,**d**) Female forewing size.

The pupation rate (Figure 6a,b), eclosion rate (Figure 6c,d), and survival rate (Figure 6e,f) in response to the radioactive caesium concentration were examined in a wide range of radioactivity levels up to H6. There was no significant difference in the Kruskal-Wallis test and no correlation in the Spearman correlation analysis. The pupation rate showed a trend of a small decrease, but it was not statistically significant. The male forewing size (Figure 7a,b) and the female forewing size (Figure 7c,d) were also examined, but no significant change was observed.

### 3.5. Direct Comparison of Equivalent Doses

**Figure 8:**
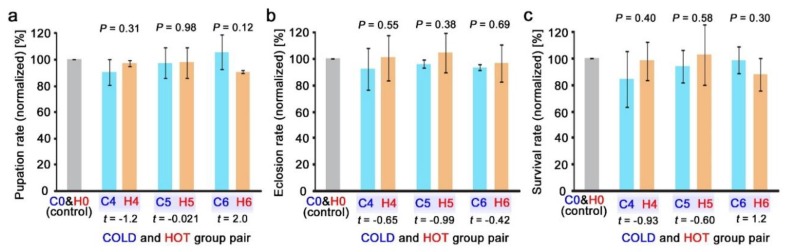
Comparisons of the effects of nonradioactive and radioactive caesium chloride. C0 and H0 were set at 100%, and the normalised values were compared between the cold (*n* = 3) and hot (*n* = 3) results. *P*-values were obtained using a Student’s *t*-tests (*df* = 4 in all combinations). (**a**) The pupation rate (%). (**b**) The eclosion rate (%). (**c**) The survival rate (%).

The same caesium concentrations were used in the nonradioactive and radioactive systems at three concentration levels, hence the results from these levels were directly compared between the two systems. No significant difference was obtained in the pupation rate (Figure 8a), eclosion rate (Figure 8b), and survival rate (Figure 8c) using a Student’s *t*-test.

## 4. Discussion

The present study examined the possible effects of nonradioactive (cold) and radioactive (hot) caesium chloride on the pale grass blue butterfly using an improved artificial diet. The improved diet attained high levels of the normalised survival rates that were comparable with those of the natural diet, despite using relatively small amounts of *Oxalis* leaves. The original survival rate (before normalisation) for the improved diet was not very high, mostly less than 80%, as observed in the control groups C0 and H0, but these results were likely because the larvae were reared in small dishes within an airtight container to prevent water evaporation from the small patches of artificial diet (see Materials and Methods). This experimental setup (i.e., double containers) was not completely ideal for larval development, probably because of high humidity inside the dishes, but was required to maintain the water quantity of the diet (and hence caesium concentrations) for the quantitative evaluation of the amount of caesium consumed by the larvae and to secure operational safety for experimenters because of the high dosage of radioactive caesium. The forewing miniaturization that was observed in this study and also in the previous study [43] is likely due to a smaller amount of consumption in the artificial diet than in a natural diet (*Oxalis* leaves). The experimental groups were run simultaneously side by side with the control groups (C0 and H0); therefore, our experimental system was satisfactory for toxicological examinations. Although small-sized adults were obtained in this rearing system with the artificial diet, as shown in Figure 1C, this did not harm the quality of the experimental system, either; small size adults have been known in entomological studies with artificial diets [43], although this fact indicates a room for further improvement. Furthermore, we experimentally confirmed that caesium radioactivity was indeed present in the bodies of the prepupae, and the proportion of the radioactivity retained in the bodies among the whole ingested radioactivity did not change much in the range from H1 to H6.

Having mentioned the validity of the rearing system, we failed to obtain any significant toxic effects in this study. The butterfly was tolerant of both cold and hot caesium (and its decay product) in the ranges tested. If anything, the pupation rate tended to decrease in response to the radioactivity concentration of caesium, but this was statistically insignificant. We were unable to obtain a half lethal dose (LD_50_) in this study. However, higher concentrations are not relevant to the comparison with the field-based results of the previous internal exposure experiments. Moreover, higher concentrations of hot caesium are technically demanding to handle safely. We, therefore, did not pursue higher concentrations and concluded that the toxic effects of cold and hot caesium require more than the levels of C11 and H6, respectively.

The low toxicity of the cold caesium in the present study may be compared with toxicological data from other animals. The mouse oral LD_50_ of caesium chloride is reported to be 2300 mg/kg (body) according to Johnson et al. [46] (in reference to Khosid (1967)), and the acute chemical toxicity in rats is also similarly low [47]. These LD_50_ levels roughly correspond to the C9 level in the present study, and the maximum C11 level in the present study was 4.68 × 10^3^ g/kg (larva). Thus, the pale grass blue larvae are much more tolerant of chemical caesium than these mammals. However, nonradioactive caesium is known as a dose-dependent inducer for chromosomal aberrations when administered in high doses in mice [48,49]. It would be interesting to know if similar cytological changes may be found in the pale grass blue butterfly at high doses.

Nonradioactive caesium is widely distributed on earth and is present in the bodies of many, if not all, organisms as a trace element [50]. The caesium contents of plants in nature varied from 0.002 to 1 ppm [50]. For example, tobacco leaves contain 0.02 ppm of caesium in dry samples [51], which corresponds to 20 μg/kg. In contrast, the chemical amount of radioactive caesium in the contaminated leaves was very small. For example, 100 Bq/kg of ^137^Cs corresponds to 31.2 pg/kg [45], meaning that the maximum caesium concentration of the field-collected leaves, 43.5 kBq/kg (leaf), corresponds to 13.6 ng/kg (leaf). Although the natural caesium content in *Oxalis* leaves is not known, it is reasonable to conclude that the amount of radiocaesium in the leaves contaminated by the Fukushima nuclear accident is negligibly small compared to that of naturally occurring caesium in plants.

Thus, because of the high caesium tolerance of the larvae and the small amount of radiocaesium in leaves, the chemical effect of radiocaesium (and its decay product ^137^Ba) on the larvae of the pale grass blue butterfly from the contaminated leaves can be considered insignificant, at least at the organismal and organ levels. Natural historically, the host plant *O. corniculata* thrives on seashores where sea salts may accumulate in and on leaves. To cope with this situation, the larvae of this butterfly may have developed an efficient salt exclusion system that makes this butterfly tolerant of several salts, including caesium chloride.

The high tolerance of insects to ionising radiation is also expected from the previous literature [52]. For example, in the silkworm moth *Bombyx mori*, wing deformation in 50% of externally irradiated individuals was obtained at 90 Gy when ^137^Cs was used as an irradiation source [53]. Considering that insect bodies are small and thus the external and internal exposures may have similar effects, this level is likely much higher than the levels used in our system, although we do not exactly know the levels of the absorbed doses for the pale grass blue butterfly in our system. It is likely that the pale grass blue butterfly is as resistant to ionising radiation as other insects. This is in contrast to the previous finding that the pale grass blue butterfly is vulnerable to radioactively contaminated leaves from the field.

It is important to stress that the maximum radiocaesium levels of the field-collected contaminated leaves that were used for the previous internal exposure experiments correspond to the level between H3 and H4. The H6 level was thus more than one hundred times as high as the maximum level of the field-collected leaves. The half abnormality dose of the previous internal exposure experiments with the field-collected leaves is 1.36 × 10^4^ Bq/kg (larva) [35], which roughly corresponds to the level of H2. Thus, the different toxicological results between the previous internal exposure experiments and the present experiments are striking. The proportion of the radioactivity retained in the bodies among the whole ingested radioactivity was independent of the ingested dose in this study, but the proportion appeared to saturate in the previous studies [29,33], suggesting that in the previous studies, radioactive caesium may be in the form of particulate matter. The field-collected leaves in the previous studies might have had numerous caesium-containing particles on the surface of the leaves, which were retained in the larval digestive tract. Radioactivity was concentrated in a small particle; therefore, it may efficiently damage intestinal cells, resulting in the high toxicity found in the previous studies [29,33]. Nonradioactive particle effects from physical and immunological damage may also occur [40,41,42,54].

To be sure, there are technical differences. In the previous internal exposure experiments, the contaminated leaves were fed to the second (and later) instar larvae. In contrast, in the present experiment, the artificial diet was given to the late third (and later) instar stages. The quality of the diets and containers were also different. However, these differences would be insufficient for explaining the large difference in the toxicological results.

On the other hand, we believe that there is a real possibility that more stringent examinations at the organismal, cellular, and molecular levels would reveal unexpected toxicological effects on the butterfly at high nonradioactive and radioactive doses. For example, we did not examine the growth rate (i.e., larval and pupal periods) in the present study. The growth rate may be an important indicator in this butterfly [29]. In aphids, the growth acceleration was observed in an ecotoxicological experiment [28]. Oxidative stress may also change the gene expression patterns of larvae, which was not examined in the present study.

Nonetheless, this study was the first case in which three types of toxicological data were collected from the same biological species: (1) field-based ecological toxicology (ecotoxicology) (including chemical and radioactive effects), which was examined previously by internal exposure experiments using contaminated leaves from the field [29,33,34]; (2) laboratory-based chemical toxicology (chemotoxicology), which was examined by feeding the larvae nonradioactive caesium in the present study; and (3) laboratory-based radioactive toxicology (radiotoxicology), which was examined by feeding the larvae radioactive caesium in the present study. In the case of ecotoxicology, the results of the internal exposure experiments were supported fully by the field sampling of butterflies for morphological examinations and by the rearing experiments for the offspring generations from the field-caught parent butterflies [29]. Furthermore, similar internal exposure experiments were performed using the white cabbage butterfly, *Pieris rapae*, under rigorous experimental conditions, indicating again high sensitivity of this species to radioactively contaminated leaves [55]. In the internal exposure experiments, the contamination levels of the leaves were evaluated based on the radioactivity concentration of caesium, but the direct cause of toxicity in the field is unlikely to be attributed solely to the direct ionising effect of caesium because no toxicity was obtained in the chemotoxicological and radiotoxicological data of caesium in the present study. Instead, other secondary causes may be in effect.

A similar insightful case is worth mentioning here. Severe and high-frequency morphological abnormalities in gall-forming aphids were reported in polluted areas after the Fukushima accident [3]. However, such morphological abnormalities were not reproduced by irradiation experiments, although a change in developmental time was detected [28]. Thus, the discrepancy between the ecotoxicological and radiotoxicological data may generally be observed in insects. This discrepancy may be called the field-laboratory paradox [42]. Based on the present results and the previous studies [29,30,31,32,33,34,35], we believe that there must be unknown mechanisms in the field that increase the toxicity of polluting radionuclides.

There may be secondary (indirect) effects of radioactive pollution mediated by ecological systems [40,41,42], which may be collectively called the field effects [42]. For example, chemical changes in the host plant leaves may be induced by radiation stress. Toxic chemicals such as reactive oxygen species (ROS) and defence chemicals may be produced in the leaves, or some metabolic pathways for essential nutrients may be blocked. Either way, the larvae that ate such leaves may exhibit mortality. Because all host plant leaves were affected in an entire contaminated area at once, butterfly larvae had no way to escape from toxic chemicals or nutritional deficiency in the field after the accident. These effects may not be observed in the laboratory-based experimental system where such ecological media are absent. The biological effects of the Fukushima nuclear accident are expected to be evaluated not only by dosimetric analysis of ionising radiation but also from the viewpoint of the field effects through ecological systems [42,56,57] together with other viewpoints [58,59,60,61] in the future.

## 5. Conclusions

Toxicological tests of nonradioactive and radioactive CsCl on the pale grass blue butterfly was performed, which revealed that this butterfly was highly tolerant not only to high chemical concentrations but also to high radioactivity concentrations. The results of the current laboratory experiments, thus, logically delineated a picture of the field-laboratory paradox. The field-laboratory paradox may be solved by the introduction of the field effects, which are field-mediated secondary effects on organisms from anthropogenic radioactive materials. Further studies on the field-effect are expected to clarify the paradox in the future.

## Appendix A

Some important steps for experimental procedures are here shown in Figure A1 so that the procedures are reproducible.
